# Treatment of neovascular age-related macular degeneration with anti-vascular endothelial growth factor drugs: progress from mechanisms to clinical applications

**DOI:** 10.3389/fmed.2024.1411278

**Published:** 2024-07-19

**Authors:** Shizhou Cheng, Shu Zhang, Mingyan Huang, Yuxuan Liu, Xunyu Zou, Xiaoming Chen, Zuhai Zhang

**Affiliations:** Department of Ophthalmology, The First Affiliated Hospital of Yangtze University, Jingzhou, China

**Keywords:** neovascular age-related macular degeneration, vascular endothelial growth factor, treatment, mechanism, clinical application

## Abstract

Neovascular age-related macular degeneration (nARMD) is an important cause of visual impairment and blindness in the elderly, with choroidal neovascularization in the macula as the main pathological feature. The onset of nARMD is closely related to factors including age, oxidative stress, and lipid metabolism. Vascular endothelial growth factor (VEGF) is an important factor contributing to nARMD as well as choroidal neovascularization and retinal leakage formation. At present, anti-VEGF therapy is the only treatment that improves vision and halts disease progression in most patients, making anti-VEGF drugs a landmark development for nARMD treatment. Although intravitreal injection of anti-VEGF drugs has become the first-line treatment for nARMD, this treatment has many shortcomings including repeated injections, poor or no response in some patients, and complications such as retinal fibrosis. As a result, several new anti-VEGF drugs are being developed. This review provides a discussion of these new anti-VEGF drugs for the treatment of nARMD.

## 1 Introduction

Age-related macular degeneration (AMD) is an ophthalmic disease involving the macular region of the retina that results in central vision loss (1, 2). The prevalence of AMD is increasing and it has become the leading cause of vision loss in older people in developed countries, affecting 10–13% of individuals aged 65 and older (3–5). The pathophysiology of AMD includes inflammatory mechanisms affecting the retina as well as oxidative stress. The disease is broadly divided into two types: dry AMD and wet AMD. Neovascular age-related macular degeneration (nARMD) is the main cause of vision loss in AMD patients (6). Treatment options have significantly evolved in recent years, greatly improving prognosis for nARMD patients. Early therapies for nARMD include laser photocoagulation, photodynamic therapy (PDT), transpupillary thermotherapy (TTT) (7), and surgery (8). At present, the gold standard therapy for nARMD is vitreous injection of anti-vascular endothelial growth factor (VEGF) drugs (8–11). However, anti-VEGF drug treatment has various limitations including the need for multiple intravitreal injections (12), the progression of macular atrophy during treatment (13), the generation of subretinal fibrosis (14), the return of visual acuity to baseline levels after 5 years of treatment (15), and poor response or non-response in some patients (16).

In this review, we systematically summarize and discuss the commonly used anti-VEGF drugs currently available for the treatment of nARMD and briefly summarize clinical trials of novel anti-VEGF drugs, highlighting potential new targets and mechanisms of action for next-generation therapies.

## 2 Mechanisms of neovascularization

Angiogenesis is a dynamic process in which new capillaries are formed on top of existing vessels in a sprouting manner. Angiogenesis is a hallmark of tissue repair, expansion, and remodeling during physiological processes such as wound healing and is tightly regulated by the body. Angiogenesis may be abnormally activated in certain pathological conditions, such as malignant tumors, atherosclerosis, chronic inflammation, and diabetic retinopathy (17–20). Notably, these diseases share various common features including the development of hypoxia or inflammation, the production of angiogenic growth factors, endothelial cell (EC) migration, proliferation and differentiation, and the regulation of vascular support cells.

**Figure 1:**
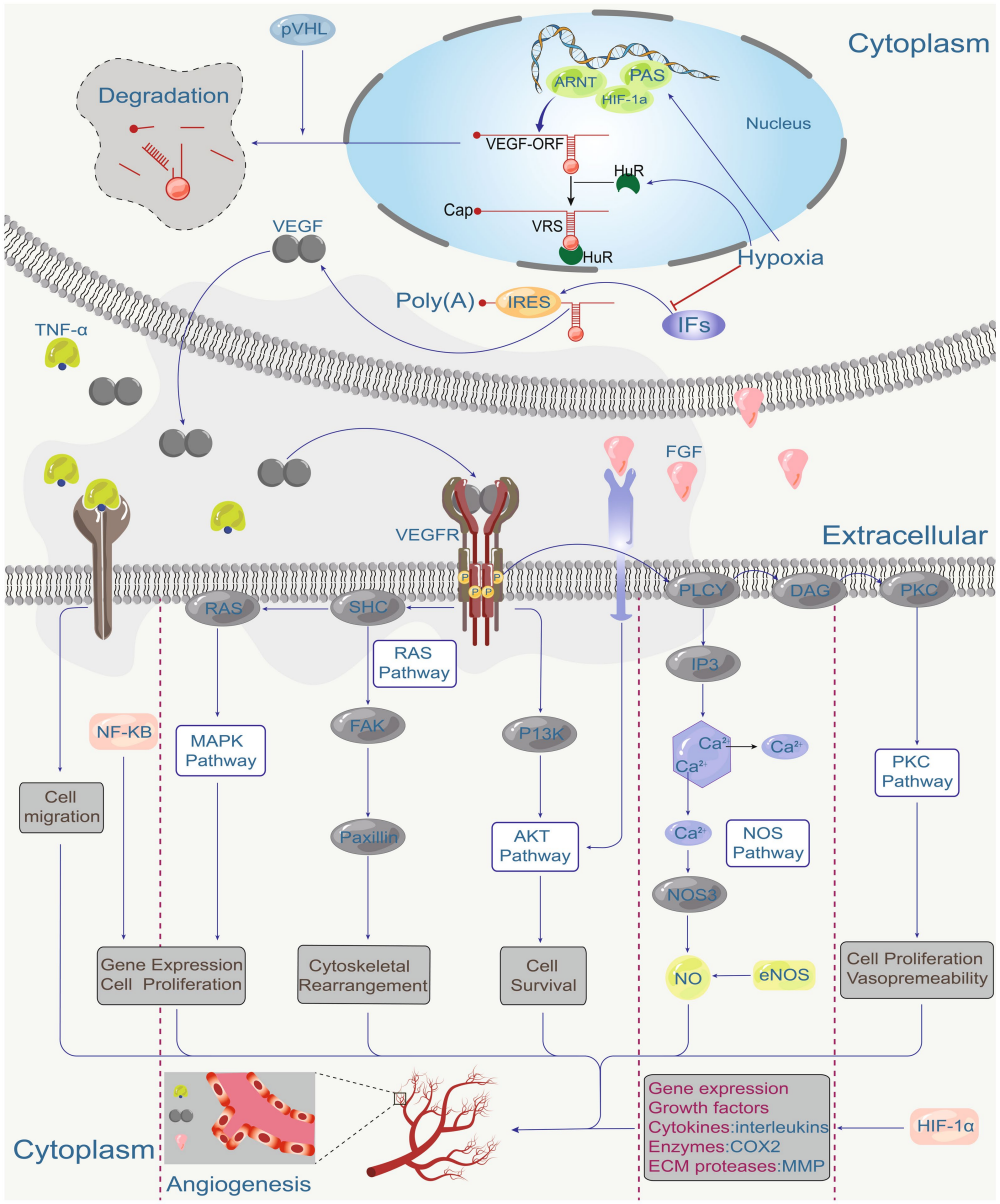
Hypoxia is the main factor regulating VEGF expression through hypoxia-inducible factor (HIF). HIP coordinates VEGF expression with other hypoxia-regulated genes, various environmental factors, and the regulation of tumor suppressor genes such as pVHL, which in turn coordinates VEGF-driven signalling. The angiogenic factor (VEGF), fibroblast growth factor (FGF), and tumor necrosis factor (TNF-α) act on receptors on endothelial cells. These receptors activate the PKC pathway, NOS pathway, AKT pathway, RAS pathway, MAPK pathway, various downstream signalling pathways (RAS, SHC, FAK, Paxillin, P13K, PLCY, IP3, DAG, PKC, NOS3, eNOS, NO), and gene expression regulators (NF-κB), ultimately directing cell proliferation, migration, survival and vascular permeability to mediate angiogenesis.

The VEGF signalling pathway plays a central role in angiogenesis (Figure 1) (21). VEGF signaling is critical for the physiological function of many tissue types and is present at angiogenic sites. After binding to tyrosine kinase receptors (VEGFRs), VEGFs are activated and form homo- or heterodimers triggering intracellular signaling cascades that stimulate endothelial cell proliferation, migration, differentiation, tube formation, and permeability control (18). VEGF/VEGFR signalling plays an important role in the pathogenesis of many diseases including cardiovascular disease, cancer, and ocular disease. For example, VEGFA promotes angiogenesis, blood-retinal barrier disruption, inflammation, and vision loss in individuals with eye diseases such as retinopathy of prematurity, diabetic retinopathy and nARMD (22–24). Other pro-angiogenic factors, such as fibroblast growth factor (FGF), platelet-derived growth factor (PDGF), placental growth factor (PIGF), and hepatocyte growth factor (HGF), play essential roles in the activation of endothelial cells (ECs) and respond to and direct the migration of ECs in the direction of pro-angiogenic stimuli to promote neoangiogenesis (18).

## 3 Pathogenesis of nARMD

**Figure 2:**
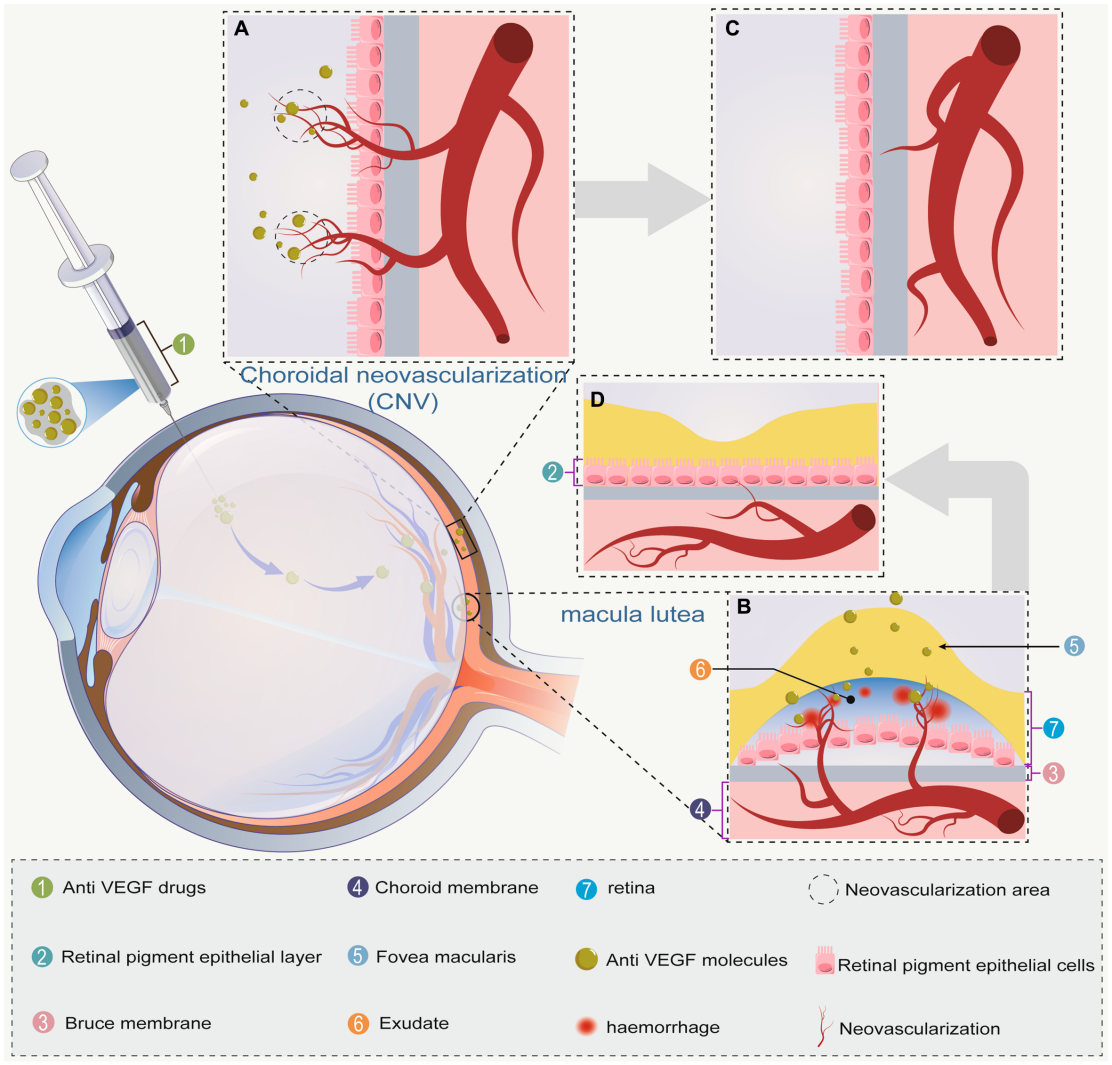
**(A)** Choroidal neovascularization breaks the vitreous membrane. The anti-VEGF drug is then injected into the vitreous cavity to reach the site of action; **(B)** neovascular macular degeneration causes macular edema via ruptured neovascularization and hemorrhage. After anti-VEGF drug injection, the drug reaches the site of action; **(C)** choroidal neovascularization gradually subsides due to the effect of anti-VEGF drugs; **(D)** choroidal neovascularization in the macula has subsided, hemorrhage has improved, and macular edema has subsided. Bold gray arrows indicate the regression of **A** → **C** and **B** → **D**.

AMD is the result of a complex interaction between environmental factors and genetic. Smoking is the most consistently identified modifiable risk factor, but dietary factors, solar insolation, and season of birth may also affect AMD incidence and progression (25, 26). nARMD is characterized by macular neovascularization (MNV) or retinal neovascularization, and is sometimes accompanied by retinal edema and retinal exudates, hemorrhages, and scarring (Figure 2). The pathogenesis of nARMD remains unclear, but is thought to relate to multiple factors such as age, genetic factors, oxidative stress, and lipid metabolism (27–29).

### 3.1 Growth factors

VEGF is regarded as a key pathogenic factor in nARMD. The main function of VEGF is to promote angiogenesis by activating cell signaling pathways. Under normal physiological conditions, this regulation is tightly controlled; however, conditions such as hypoxia, ischemia, and inflammation tilt the balance, thereby increasing VEGF expression in a way that promotes neovascularization (30). The VEGF family comprises seven members (31). VEGF receptors have different roles. Among these receptors, VEGFR2 plays a key role in wet AMD. VEGFR2 in endothelial cells is translocated to the cell surface where its tyrosine kinase can be activated by VEGF-A to induce angiogenesis and increase vascular permeability (32). Angiopoietin-2 (Ang-2) is a growth factor with roles in vascular homeostasis, angiogenesis, and vascular permeability that interacts with the Tie-2 transmembrane receptor tyrosine kinase expressed by endothelial cells. Ang-2 levels are upregulated under conditions such as hypoxia and oxidative stress. In addition to its roles in vascular leakage and abnormal changes in vascular structure, Ang-2 enhances pro-inflammatory signalling in endothelial cells. Co-expression of Ang-2 and VEGF-A has been shown to promote neovascularization in models of retinal development and retinal ischemia (33) and may play an important role in the development of nARMD (34). TGF-β has been shown to induce angiogenesis *in vivo* and to stimulate VEGF secretion from human RPE cells *in vitro* (35). In addition, TGF-β regulates endothelial cell proliferation and macrophage infiltration, as well as extracellular matrix deposition and protein hydrolysis, which activate angiogenesis and vascular remodeling (36), and plays an important role in the progression of nARMD.

### 3.2 Complement system activation

Immunohistological and proteomic analyses of postmortem tissues from AMD patients have revealed the presence of complement proteins and their activation products in vitreous warts. Furthermore, population-based genetic analyses have shown that several variants of complement proteins are associated with an increased risk of AMD (37, 38). The main role of the complement system, which is part of the innate immune system, is to recognize and remove foreign pathogens, apoptotic cells, and cellular debris. The classical, alternative, and lectin pathways are the initiating pathways for complement activation, all of which involve cleavage of complement component 3 (C3). C3 produces more C3b and C3a in the presence of lytic enzymes and further exacerbates cellular destruction. The ability to amplify alternative pathways appears critical for the development of wet AMD (39).

### 3.3 Inflammatory vesicles and inflammatory factors

Inflammatory vesicles are multiprotein complexes. A typical inflammatory vesicle complex consists of the cytoplasmic sensor protein, the adapter protein ASC (apoptosis-associated speck-like protein), and the effector protein procaspase-1. Inflammatory vesicles induce CNV via a complement-independent mechanism (40). AMD is associated with chronic inflammation, and the eyes of AMD patients show high expression of inflammation/immunity-related factors, including IL-17 receptor C, IL-17F, IL-6, etc. The retina and choroid can recruit numerous macrophages and microglia, which are distributed among the non-immune cells (RPE and Müller cells), and are involved in disease development.

### 3.4 Autophagy and other factors

Autophagy is a catabolic process. Prior studies have shown that nARMD patients have an increased number of autophagosomes in RPE cells and that autophagic dysfunction in RPE cells is involved in the pathogenesis of nARMD (41). Oxidative stress is another important pathogenetic mechanism of AMD. It is thought that oxidative stress promotes the development of MNV by stimulating VEGF production by RPE cells and other cells, as well as by inducing an inflammatory environment (28, 30).

## 4 Anti-VEGF drug therapy for nARMD

**Figure 3:**
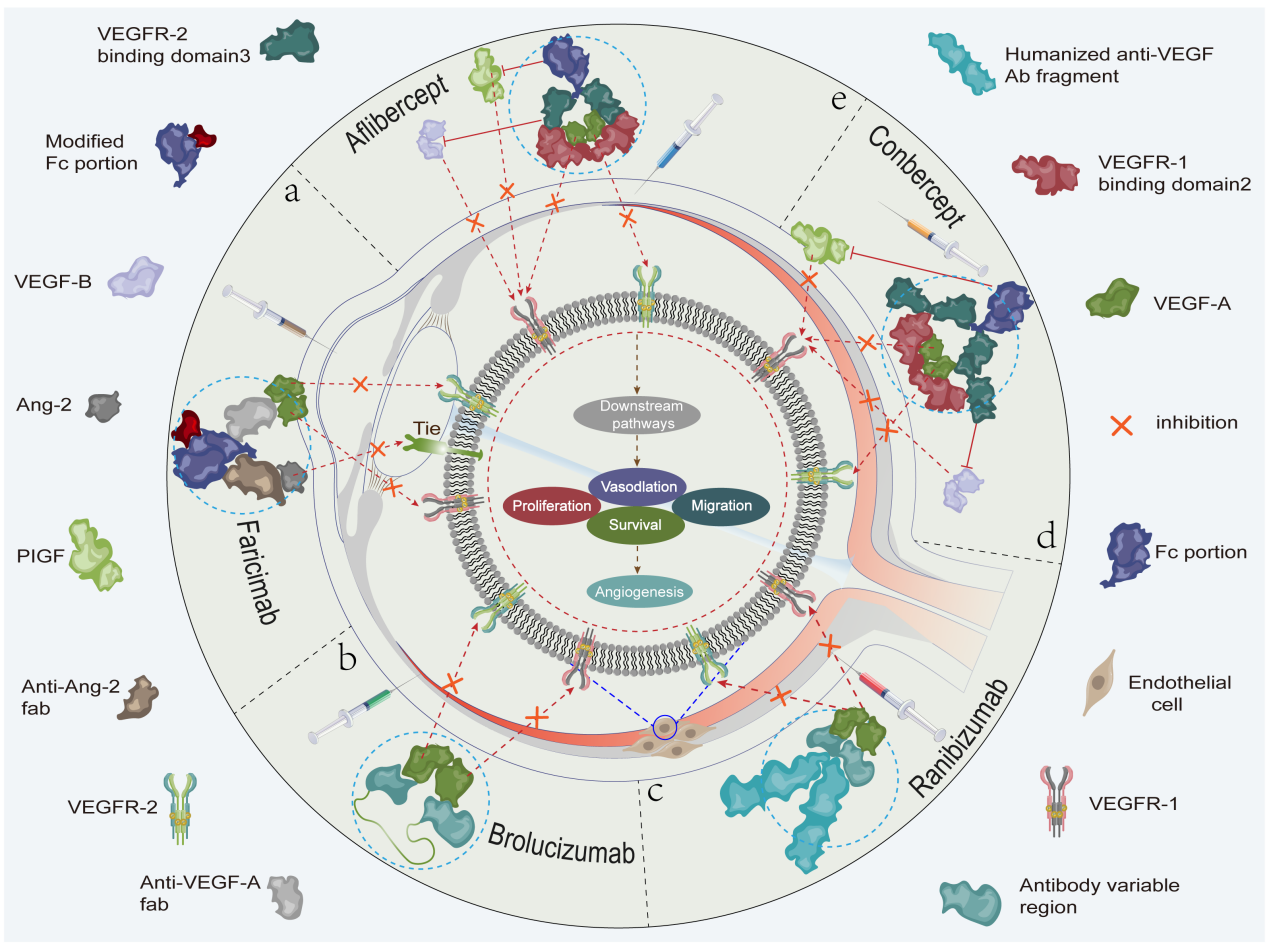
**(A)** Faricimab potently and specifically binds to and inhibits both the VEGF-A/Ang-2 pathways to stabilize blood vessels. **(B)** The brolucizumab molecular fragment (antibody variable region) binds to VEGF-A and inhibits the binding of VEGF-A to VEGFR-1 and VEGFR-2. **(C)** Ranibizumab binds to VEGF-A and inhibits the binding of VEGF-A to VEGFR-1 and VEGFR-2. **(D)** Conbercept binds to VEGF-A, VEGF-B, and PIGF to inhibit the binding of VEGF-A to VEGFR-1 and VEGFR-2, the binding of PIGF to VEGFR-1, and the binding of VEGF-B to VEGFR-1. **(E)** Aflibercept binds to VEGF-A, VEGF-B, and PIGF to inhibit the binding of VEGF-A to VEGFR-1 and VEGFR-2, the binding of PIGF to VEGFR-1, and the binding of VEGF-B to VEGFR-1. As shown, the related anti-VEGF drugs inhibit cell proliferation, migration, survival, and vascular permeability. This ultimately prevents neovascularization by inhibiting (fork symbols) the binding of vascular endothelial growth factor to the corresponding receptors, thereby blocking activation of the relevant signaling pathways.

VEGF is an important regulator of neoangiogenesis and leakage in MNV, which contribute to the proliferation of vascular endothelial cells and induce neovascularization and increased vascular leakage (42). Recent studies have shown that the blockade of VEGF expression induces the onset of vascular remodeling while contributing to regression of immature neovascularization. Given the important role of VEGF in the generation and maintenance of neovascularization, anti-VEGF drugs are currently the first-line choice for clinical treatment of nARMD (8–10). Treatment of nARMD can effectively abate choroidal neovascularization, macular edema, and hemorrhage to prevent the occurrence of serious complications (Figure 2). At present, the most commonly used anti-VEGF drugs include ranibizumab, aflibercept, conbercept, faricimab, and brolucizumab (Table 1) (55). Conbercept and aflibercept, which are fusion proteins formed by recombination of the binding domains of the extracellular regions of human VEGF-1 and VEGF-2 with the Fc segment of human immunoglobulin, mainly function by binding to VEGF-A and placental growth factor (PIGF). Ranibizumab is a humanized IgG1 monoclonal antibody fragment that inhibits VEGF-A activity by directly binding to it. Brolucizumab is a humanized monoclonal single-chain antibody fragment (scFV) that binds to VEGF-A to prevent its interaction with VEGF-1 and VEGF-2 receptors, thereby inhibiting neovascularization. Faricimab is potent and specific in binding and inhibiting both the VEGF-A/Ang-2 pathways, stabilizing the vasculature and reducing leakage more than inhibiting either pathway alone (Figure 3). In addition although bevacizumab has been shown to be effective in the treatment of nARMD (56, 57), the drug is not yet approved for intravitreal injection, there is no dedicated intravitreal formulation, and the concentration needs to be adjusted at the time of use, which increases the likelihood of contamination and the risk of endophthalmitis, and therefore remains a controversial therapeutic option (58).

**Table 1:** Molecular structures and applied dosages of different anti-EGF drugs.

Veterinary drug	Characteristic	Molecular mass (kDa)	Clinical dose(mg)	Retinal exposure concentration ratio	Peak retinal exposure time (h)	Intravitreal half-life (d)	Ref
Conbercept	Fusion protein	143	0.5	-	6–12	3.70	(7, 43, 44)
Aflibercept	Fusion protein	97–115	2.0	-	24	3.63	(45)
Ranibizumab	Ab	≤48	0.5	1	6	2.60	(46–48)
Brolucizumab	scFv	26	6.0	2.2–3.1	1–6	2.40	(21, 49–51)
Faricimab	Dual antibody	-	6.0	-	48	7.50	(52–54)

### 4.1 Recombinant fusion protein-based anti-VEGF drugs

#### 4.1.1 Conbercept

The novel anti-VEGF conbercept is a fusion protein with a relative molecular mass of 143,000 composed of immunoglobulin-like region 2 (FLt-1) of human VEGFR1 and immunoglobulin-like regions 3 and 4 of VEGFR2 and fused to the Fc segment of human IgG1 to give it a high affinity and long half-life; the extracellular domains of the VEGFR2 of conbercept can significantly reduce neovascularization and, at the same time, enhance the binding of conbercept to VEGF (7, 43, 44). In the phase 2 clinical trial of conbercept for the treatment of nARMD (AURORA), visual acuity and anatomical restoration were maintained or further improved after different dosing regimens with different dosages (CONBERCEPT 0.5 versus 2.0 mg monthly or PRN) (59). In the phase III clinical trial (PHOENIX), the conbercept injection group received a monthly intravitreal injection (0.5 mg) for 3 months followed by quarterly injections until month 12, while the control group received a monthly sham injection for 3 months, followed by a quarterly injection of conbercept (0.5 mg) until month 12. At 3 months, the mean increase in best corrected visual acuity (BCVA) was 9.20 letters in the conbercept group versus 2.02 letters in the control group (*p* < 0.001); at 12 months, the mean increase in BCVA was 9.98 letters in the conbercept group versus 8.81 letters in the control group (*p* = 0.64). These findings indicate that monthly intravitreal conbercept injection for 3 months, followed by administration every 3 months, resulted in improved and sustained visual acuity and is effective for the treatment of AMD, whereas the other anti-VEGF drugs did not maintain a similar clinical effect with quarterly administration (7). A meta-analysis of the efficacy and safety of conbercept for the treatment of nARMD found no difference in clinical efficacy between conbercept and ranibizumab and that serum VEGF levels were lower in the conbercept group than in the ranibizumab group (44, 60). Furthermore, conbercept was well tolerated intravitreally with no serious adverse events (AEs) reported in the PHOENIX study: the most common ocular AEs were elevated intraocular pressure and subconjunctival hemorrhage, and the large molecular size of conbercept limits the permeability of the blood-ocular barrier and reduces systemic exposure (7). As a new generation drug, conbercept has the advantages of multi-targeting, high affinity, long half-life, and low levels in serum. Large-sample, multi-center clinical studies are needed to further evaluate the long-term efficacy and safety of conbercept and to develop individualized patient regimens, testing the need to maximize visual gain and minimize treatment frequency.

#### 4.1.2 Aflibercept

Aflibercept is a recombinant protein comprising two protein sequences of the VEGF receptor (VEGFR-1 and VEGFR-2) and the Fc portion of immunoglobulin G1. Aflibercept binds with high affinity to all isoforms of VEGF-A as well as to VEGF-B and placental growth factor (PLGF), inhibiting the downstream signaling mediated by these receptors. *In vitro*, the equilibrium dissociation constants (KD) of aflibercept with human VEGF-A121 and -A165 isoforms are 0.36 and 0.49 pmol /L, respectively (45). Since its approval by the U.S. FDA in November 2011 (61), aflibercept has become the initial therapeutic drug of choice for nARMD with studies showing that intravitreal injection of aflibercept effectively improves BCVA and reduces central macular thickness (CMT) in nARMD patients (62–66).

Currently, the mainstream treatment regimens are 3 + PRN (*pro re nata*) and treatment-and-extend (T&E). The 3 + PRN regimen consists of one injection per month for the first 3 months of treatment, followed by monthly follow-up and re-injections as indicated (67). The T&E regimen consists of a gradual extension of treatment and follow-up to determine the longest interval between treatment and follow-up for individual patients (68). The T&E regimen has been shown to be effective at reducing the number of injections per month during the first 3 months of treatment. Both the PRN and T&E regimens are clinically designed to reduce the burden of anti-VEGF therapy and to stabilize BCVA. The T&E regimen is the preferred option among Spanish retina specialists who believe that, because of the longer duration of drug action in the body of aflibercept and fitch, the regimen is suitable for routine clinical use (69). A UK panel of experts also recommends the T&E regimen, setting specific criteria based on visual acuity and retinal morphologic features shown by optical coherence tomography to achieve longer injection intervals, fewer injections, and maintenance of stable visual acuity. The panel suggests that the T&E regimen has a stronger long-term effect than the PRN regimen for predicting future demand for nARMD treatment (70). The T&E regimen has become a standard treatment option for ARMD with more experts choosing this regimen for the treatment of nARMD with aflibercept (Table 2).

**Table 2:** Comparison of the advantages and disadvantages of the PRN and T&E programs.

PRN program	T&E program
Passive treatment program	Active treatment program
Unpredictable injection schedule	Predictable injection schedule
Regular monthly follow-up visits are burdensome for doctors and patients	No follow-up visits between injections, less burden on doctors and patients
Already in clinical practice and less difficult to implement	The new program process requires learning and is difficult to implement
Good long-term outcomes can only be achieved when rigorous testing and re-treatment criteria are adopted	Good long-term visual outcomes

PRN: pro re nata; T&E: treat-and-extend.

Aflibercept, like other anti-VEGF drugs, can cause subconjunctival hemorrhage, intraocular infection, ocular pain, increased intraocular pressure, cataracts, and posterior vitreous detachment. A randomized, double-blind intervention study found that both treatments significantly increased BCVA and decreased CMT in patients, and that the 1.25 mg/0.05 mL and 2 mg/0.08 mL aflibercept regimens had similar safety profiles without any major unexpected AEs (71). Other studies have reported that tachycardia occurs in a small number of patients with nARMD after repeated aflibercept injection, particularly those with subretinal pigment epithelial lesions and no intraretinal edema (72). In a study of aflibercept for the treatment of nARMD, 8 patients (12.7%) developed early tachycardia or shortness of breath, which is an important clinical problem in patients receiving long-term anti-VEGF therapy (73). Patients’ macular function continued to improve during intravitreal injections of aflibercept and peripapillary retinal cone cell function declined after multiple treatments, suggesting that aflibercept may have adverse effects on normal peripapillary retinal function in the macular area (74). From the perspective of current clinical applications, aflibercept is safe. Although adverse reactions can be treated and recovered within a short period of time, caution is warranted when using aflibercept and effort should be made to increase the number of patients in trials and carry out longer-term follow-up.

### 4.2 Monoclonal antibody-based anti-VEGF drugs

#### 4.2.1 Ranibizumab

Ranibizumab is a second-generation recombinant humanized monoclonal antibody to Fab fragments with a relative molecular mass of 48,000 and high binding affinity to various isoforms of VEGF-A (VEGF165, VEGF121, and VEGF110) (46–48). Ranibizumab has been proven to be safe and effective in several clinical trials, including MARINA, ANCHOR, FOCUS, PIER, and PrONTO. Ranibizumab can effectively improve retinal morphology, reduce choroidal neovascularization, reduce retinal thickness, and improve patients’ visual acuity (75–78). However, a 7-year follow-up study found that vision stabilized in about half 1/2 of eyes compared to baseline vision, but that 1/3 of eyes had a loss of 15 letters or more and 98% of eyes had macular atrophy and photoreceptor cell damage (16). There are rare reports of localized adverse reactions including endophthalmitis, uveitis, vitreous hemorrhage, primary retinal detachment, retinal tears, and lens damage, but more common reports of transient elevation of intraocular pressure. Systemic adverse reactions include aortic aneurysm, atrial fibrillation, carotid artery stenosis, coronary artery disease, falls, femur fracture, gastrointestinal bleeding, and systemic immune system reactions (79). For current clinical treatment of nARMD, ranibizumab is the main first-line anti-VEGF drug.

#### 4.2.2 Brolucizumab

Brolucizumab is a humanized monoclonal single-chain antibody fragment (scFV) produced by *Escherichia coli* through DNA recombination technology that shows a high affinity for all three major isoforms of VEGF-A (VEGF110, VEGF121, and VEGF165) and prevents their interactions with the VEGF-1 and VEGF-2 receptors, thus inhibiting neovascularization and decreasing vascular permeability (Fifure 2) to play a role in the treatment of nARMD (21, 49, 50).

At present, brolucizumab is the smallest molecular weight (26 kDa) VEGF conjugate and has a molecular weight less than 1/4 that of aflibercept (115 kDa), less than 1/5 that of conbercept (143 kDa), and approximately 1/2 that of ranibizumab (48 kDa). Brolucizumab binds to VEGF-A at a ratio of 2:1. The amount injected into the intravitreal cavity in 0.05 mL can reach 6 mg. ml of vitreous cavity to achieve a volume of 6 mg (51), the molar concentration of brolucizumab is 12, 66, and 22 times higher than that of aflibercept, conbercept, and ranibizumab, respectively. It is because of brolucizumab’s low molecular weight and high molar concentration that it can effectively penetrate the retina and choroid. In studies with rabbits, brolucizumab was exposed at concentrations 2.2-fold higher than that of ranibizumab in the retina and 1.7-fold higher than that of ranibizumab in the retinal pigment epithelial/choroidal layer (80). Ranibizumab has a maximum peak time of 6 h in the monkey vitreous cavity and a half-life of 2.6 days, aflibercept has a maximum peak time of 24 h in a rabbit model and a half-life of 3.63 days, conbercept has a maximum peak time of 6–12 h in the rabbit vitreous cavity and a half-life of 3.7 days, and brolucizumab has a maximum peak time of 1–6 h in the monkey retina and a half-life of 2.4 days (81–83). Due to these properties and the absence of the Fc domain, brolucizumab is cleared rapidly and systemically (5.6 ± 1.5 h). As retinal exposure is maintained at a high level, this reduces the risk of systemic adverse effects while maintaining a durable therapeutic effect. The mean half-lives of brolucizumab, ranibizumab, and aflibercept in the vitreous cavity are reported to be 56.8, 62, and 53 h, respectively (84).

The incidence of aseptic intraocular inflammation (IOI) after brolucizumab treatment is up to 4.6% (85), which was slightly higher than that of other anti-VEGF agents (0.3–2.9%) (86). Pre-existing IOI and simultaneous bilateral injections have been suggested as risk factors for the development of IOI after brolucizumab injection (87). Mukai et al. (88) reported that advanced age, female sex, and diabetes are also risk factors for IOI after injection. Other studies have found that IOI is somewhat self-limiting, with some cases resolving on their own without treatment (86). The cause of IOI occurrence may be the small molecular weight of brolucizumab or that the epitope is not recognized by the immune system after intraocular exposure. Secondary IOI may be due to a type III hypersensitivity reaction. Early recognition of inflammation due to an immunogenic reaction and prompt glucocorticoid treatment are thus essential when applying brolucizumab to patients with concomitant endophthalmitis. Brolucizumab has been widely used for the treatment of nARMD since it was approved by the FDA in 2019. The available clinical data indicate that brolucizumab has a favorable safety profile and provides a new pathway for clinical treatment of nARMD compared with other anti-VEGF drugs.

### 4.3 VEGF-A/Ang-2 dual-targeting based anti-VEGF drugs

#### 4.3.1 Faricimab

Co-expression of Ang-2 and VEGF-A has been shown to accelerate CNV formation. On the one hand, Ang-2 competitively inhibits the Ang-1/Tie2 pathway, disrupting the interaction between vascular endothelial cells and pericytes, returning endothelial cells to and activated state and, simultaneously, attracting further aggregation of angiogenic factors, such as VEGF, to the endothelial cells to cause endothelial cell outgrowth, neovascularization, and vascular exudate (52). On the other hand, unlike the stable expression of Ang-1, Ang-2 is inducible and VEGF stimulates Ang-2 secretion by Weibel Palade vesicles, which reduces the stability of the vessels and ultimately leads to the formation of CNV (53). Faricimab is a humanized bispecific IgG1 antibody that acts through dual pathway inhibition by binding to and neutralizing VEGF-a and Ang-2. It has been show recently that Ang2, play a crucial component of the Ang/Tie pathway, plays a multifaceted role in vascular homeostasis, influencing vascular permeability and participating in neoangiogenic and proinflammatory processes. For these reasons, Faricimab may have a specific influence on choroidal flow signal (89, 90). Studies of mouse CNV models have shown that, although both anti-Ang-2 and anti-VEGF-A/Ang-2 treatments reduce CNV count and vascular exudation, combined anti-VEGF-A/Ang-2 treatment is more effective (54). Significant reduction in macular retinal thickness from baseline levels after treatment with faresimab has been reported in a clinical trial of nARMD patients (91, 92). Intravitreal faricimab is generally well tolerated in clinical trials of patients with nAMD or DME with an adverse event and safety profile comparable to that of aflibercept (91, 92).

### 4.4 Novel anti-VEGF drugs

Several major anti-VEGF drugs still require long-term treatment and a large proportion of patients are unresponsive or non-responsive. Therefore, it remains important to investigate smaller molecular weight and longer half-life anti-VEGF drugs to prolong the treatment interval and to explore new targets to improve patient compliance and reduce the risk of vitreous injection, Novel anti-VEGF drugs currently under investigation in clinical trials are summarized in Table 3.

**Table 3:** Novel anti-VEGF drugs for nARMD currently in clinical trials.

Drug	Mechanism of action	R&D progress	Ref
Abicipar pegol	Anti-VEGF molecules based on the DARPin family	Phase III clinical completion	(93)
OPT-302	Anti-VEGF-C and VEGF-D	Phase II b clinical completion	(94)
KSI-301	Biopolymers that specifically bind VEGF	Phase Ib clinical completion	(95)
RGX-314	The anti-VEGF Fab structural domain neutralizes VEGF activity	Phase II clinical	(55)
Fovista	Anti-PDGF-B	Failed Phase III trial	(96)
X-82	Anti-VEGFA/PDGFR in oral dosage form	Phase II clinical completion	(80)
IBI302	VEGF/complement dual-target specific recombinant fully human fusion protein	Phase II clinical	(97)
GB102	A dual tyrosine kinase inhibitor with dual action on VEGFR-2 and PDGFRβ agents	Phase IIb clinical completion	(95)
LHA510	VEGF-A inhibitors in eye drop formulations	Phase II clinical	(97)
9 MW0211	Anti-VEGF-A	Phase III clinical	(98)
ADVM-022	Anti-VEGF-A and PGF	Phase II clinical	(99)

## 5 Concluding remarks and future perspectives

Overall, VEGF therapy is effective for saving or improving vision in many patients with nARMD and is the main first-line option for treatment of nARMD at present and for some time to come. VEGF is a protective growth factor that is compensatorily produced by the body. The physiological effects of VEGF are suppressed during anti-VEGF treatment, which may cause retinal atrophy, retinal pigment epithelial (RPE) tears, systemic adverse effects, and related problems. How to improve the treatment strategy to minimize such adverse effects is a major challenge for anti-VEGF therapy in the future.

In the treatment of nARMD, repeated intravitreal injections may eventually cause complications, poor treatment adherence, and a significant burden for patients and healthcare systems. With the emergence and development of new anti-VEGF drugs, the drawbacks of this therapy may be remedied in the near future, bringing new hope to patients. Notably, the dosage and method of injection of new anti-VEGF drugs should be fully evaluated at this stage, and the safety, tolerability, and efficacy of alternative or complementary anti-VEGF therapies warrant further investigation. In addition, basic research on the formation and regulation of MNV, retinal neuronal apoptosis and regeneration, and stem cell transplantation should be expanded to further improve prevention and treatment of the disease.

## Author contributions

SC: Software, Writing – original draft, Writing – review & editing, Data curation, Formal analysis, Project administration, Resources. SZ: Data curation, Formal analysis, Writing – original draft. MH: Validation, Writing – original draft. YL: Software, Project administration, Writing – original draft. XZ: Investigation, Writing – original draft. XC: Writing – review & editing. ZZ: Writing – review & editing.
